# Application of Direct Thermal Desorption–Gas Chromatography–Mass Spectrometry for Determination of Volatile and Semi-Volatile Organosulfur Compounds in Onions: A Novel Analytical Approach

**DOI:** 10.3390/ph16050715

**Published:** 2023-05-08

**Authors:** Ana V. González-de-Peredo, Mercedes Vázquez-Espinosa, Estrella Espada-Bellido, Marta Ferreiro-González, Ceferino Carrera, Miguel Palma, Gerardo F. Barbero

**Affiliations:** Department of Analytical Chemistry, Faculty of Sciences, Wine and Agrifood Research Institute (IVAGRO), University of Cadiz, Agrifood Campus of International Excellence (ceiA3), 11510 Puerto Real, Spain

**Keywords:** *Allium cepa* L., direct thermal desorption–gas chromatography–mass spectrometry, multi-response optimization, onion, organosulfur compounds

## Abstract

The population is now more aware of their diets due to the connection between food and general health. Onions (*Allium cepa* L.), common vegetables that are minimally processed and grown locally, are known for their health-promoting properties. The organosulfur compounds present in onions have powerful antioxidant properties and may decrease the likelihood of developing certain disorders. It is vital to employ an optimum approach with the best qualities for studying the target compounds to undertake a thorough analysis of these compounds. In this study, the use of a direct thermal desorption–gas chromatography–mass spectrometry method with a Box–Behnken design and multi-response optimization is proposed. Direct thermal desorption is an environmentally friendly technique that eliminates the use of solvents and requires no prior preparation of the sample. To the author’s knowledge, this methodology has not been previously used to study the organosulfur compounds in onions. Likewise, the optimal conditions for pre-extraction and post-analysis of organosulfur compounds were as follows: 46 mg of onion in the tube, a desorption heat of 205 °C for 960 s, and a trap heat of 267 °C for 180 s. The repeatability and intermediate precision of the method were evaluated by conducting 27 tests over three consecutive days. The results obtained for all compounds studied revealed CV values ranging from 1.8% to 9.9%. The major compound reported in onions was 2,4-dimethyl-thiophene, representing 19.4% of the total area of sulfur compounds. The propanethial S-oxide, the principal compound responsible for the tear factor, accounted for 4.5% of the total area.

## 1. Introduction

The *Allium* genus includes the onion, a vegetable that holds significant value both in terms of its economic and nutritional contributions [1]. The use of onions in food, including as seasoning in many dishes, has been prevalent in most countries for hundreds of years [2]. However, onions are not only used in culinary contexts but also for medical purposes [3]. Many studies have reported that onion consumption helps to prevent the occurrence of several illnesses, such as inflammatory diseases [4], cancer [5], diabetes [6], and neurological disorders [7]. These biological effects are largely associated with the chemical components of onions, especially organosulfur and phenolic compounds [8]. Regarding the latter, different studies carried out by our research group have demonstrated the high levels of flavonols and anthocyanins in onion bulbs [9,10,11]. In addition, these compounds are responsible for the color of the bulb and have strong antioxidant activity. On the other hand, organosulfur compounds can make up as much as 5% of an onion’s dry weight. They are responsible for the flavor and aroma of onions and have powerful antioxidant properties [12]. Nrf2, nuclear erythroid 2-related Factor 2, is a key factor that plays a vital role in protecting cells against oxidative stress and inflammation [13]. It has been shown that activation of this transcription factor can have a significant impact on various diseases, including renal, pulmonary, cardiovascular, neurodegenerative diseases, and cancer [14,15]. Likewise, antioxidant compounds, such as organosulfur compounds, can increase the activity of Nrf2, thus contributing to the activation of the cellular antioxidant defense system.

S-alk(en)yl cysteine sulfoxides (SCs), R-SO-CH_2-_CH(NH_2_)COOH, are the primary odorless, non-volatile organosulfur compounds found in onions and are kept in the cytoplasm of whole bulbs [16]. When onions are damaged by cutting, cooking, or chewing, the cell tissue ruptures and SCs are released, thus coming into contact with the enzyme alliinase (S-alk(en)yl-L-cysteine sulfoxide lyase) [17]. This release triggers enzymatic hydrolysis of SCs, thus generating pyruvate, ammonia, and sulfenic acids (R-S-OH) [18]. These highly reactive sulfenic acids are rearranged causing thiosulfinates (R-S-SO-R’), which in turn react with each other or with other sulfenic acids, thus leading to more stable forms such as thiosulfonates (R-SO_2_-S-R’), monosulfides (R-S-R’), disulfides (R-S-S-R’), and trisulfides (R-S-S-S-R’), as well as to other thiosulfinates [19]. This heterogeneous mixture of aromatic organosulfur compounds is a consequence of both the nature of the -R and -R’ groups of their precursors (i.e., SCs) and their proportion in onion bulbs [20]. For example, after all these fast and reactivating reactions, the S-*trans*-prop-1-enyl cysteine sulfoxide (isoaliin, CH_3_-CH=CH-SO-CH_2_-CH(NH_2_)COOH), which is the main SC of the common onion, generates propanethial S-oxide (CH_3_-CH_2_-CH=SO); this is very interesting because it is responsible for the irritation of the eyes (usually generating tears) when cutting an onion [21]. Therefore, in *Allium* vegetables, SCs serve as the precursor to a remarkable array of sensory and health-promoting active chemicals [22].

Given the importance of organosulfur compounds, their content in onions must be known to select and develop cultivars with high nutraceutical value for the food and pharmaceutical industry [18]. A range of techniques has been employed to examine these compounds, ranging from basic semi-quantitative screening to state-of-the-art chromatographic methods. These methods may be categorized as either direct or indirect [22]. Direct methods determine SCs before their enzymatic decomposition, and indirect methods determine the products obtained after enzymatic conversion [23]. The profile study of these compounds, which are known as secondary aroma compounds [8], is very important, as they are responsible for the flavor and biological qualities of onions. In addition, many studies have shown that the concentration of organosulfur compounds in the volatile and semi-volatile fractions of onions affect the antibacterial activity of sulfides [24].

Frequently, trace amounts of volatile and semi-volatile organic compounds (VOCs and SVOCs) can be found in fruits and vegetables, so effective extraction techniques and sensitive methodologies are required to properly characterize them [25]. In terms of analytical methodologies, gas chromatography combined with mass spectrometry (GC-MS) is a useful tool to characterize the volatile and semi-volatile organosulfur composition of *Allium* [8,26,27,28]. Regarding the extraction technique, conventional solvent extraction requires a high volume of solvent, as well as consuming a lot of time and money [29]. As a result, many solvent-free sample preparation methods have been developed. Solid-phase microextraction (SPME) is one of the most widely used due to its versatility, simplicity of operation, availability of many coating materials, and its robustness [30]. However, the competition for adsorption/absorption sites caused by the fiber’s low surface capacity could hinder the extraction of some VOCs and SVOCs [31]. The direct thermal desorption (DTD) method stands out as an exceptional alternative to SPME due to its capacity for a large sorbent phase. Unlike normal thermal desorption (TD), which focuses on gaseous samples, DTD is specifically designed for solid samples. Instead of using sorbents to absorb the sample, the TD tube is directly filled with the solid material, which eliminates the need for sample pre-treatment since analytes are directly desorbed at the appropriate temperature [32]. Additionally, this technique is renowned for its higher final sensitivity and lower detection limits [33,34]. However, for onions, the use of DTD or TD coupled with GC-MS has not been previously explored to analyze the profile of their organosulfur compounds.

Although DTD is a versatile preconcentration technique for GC-MS, various parameters such as the sample amount, the desorption tube temperature, or the trap heat temperature could affect the extraction. Therefore, it is crucial that the DTD method has the most suitable characteristics for the compounds to be analyzed. The use of experimental designs (DOEs) combined with the response surface methodology (RSM) is a widely adopted strategy for optimizing methodologies for the analysis of bioactive compounds. This approach enables the determination of the optimal values of the factors that minimize or maximize the response or achieve a specific goal [35]. 

Therefore, this work aims to develop and validate a direct thermal desorption–gas chromatography–mass spectrometry (DTD-GC-MS) method for the simultaneous determination of organosulfur compounds in onions using both a BBD and an RSM. This combination could allow the relationship between factors and their response to be fully understood, as well as methods to be systematically and effectively optimized. Likewise, the DTD-GC-MS method proposed in this study could be used by laboratories, researchers, and companies to better understand the organosulfur onion profile and select onion cultivars with the best nutraceutical value or sensory characteristics.

## 2. Results and Discussion

### 2.1. Qualitative Analyses

To have an overview of the profile of compounds present in onions, qualitative analyses were performed under unoptimized conditions as follows: 40 mg of onion sample; tube heating at 150 °C for 600 s; a trap temperature heating at 265 °C for 180 s. Table 1 shows the compounds divided into families.

**Figure 1:**
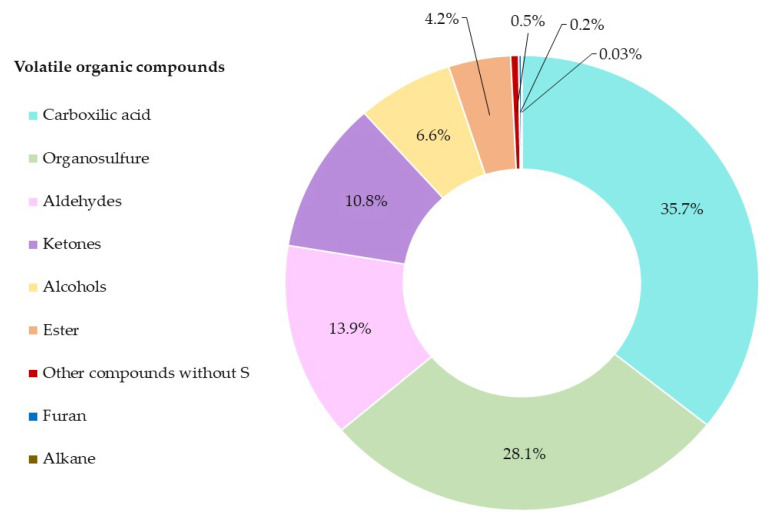
Distribution of the fifty-one compounds. Each color shows the percentage composition of the fifty-one VOCs and SVOCs following the normalization method of the chromatographic peak areas.

A total of fifty-one VOCs and SVOCs were tentatively identified, according to the Wiley Library, with a matching factor greater than 80%. Among these compounds, twenty were organosulfur compounds, and the remaining were eight aldehydes, three carboxylic acids, six alcohol, five ketones, five esters, two furans, one alkane, and one carbon dioxide. The distribution of the VOCs and SVOC in the red onion is graphically represented by a pie chart (Figure 1).

Carboxylic acids (35.7%), organosulfur (28.1%), and aldehydes (13.9%) accounted for more than 77.6% of the composition of red onion. This information agreed with the data reported by other authors who indicated that red onion is mainly characterized by these three families of compounds [27]. 

As for aldehydes, 2-methyl-2-pentenal was produced by the sequential transformation of 1-propenyl sulfenic acid into thiopropanal-S-oxide [36,37]. In addition, the condensation of propanal and acetaldehyde could produce (E)-2-methyl-2-butenal, which in turn could be reduced to 2-methyl-butanal [38]. Moreover, some aldehydes characteristics of heated onions were detected because of the temperatures applied during DTD. Likewise, acetaldehyde, propanal, 2-methyl-butanal, and 2-methyl-propanal have been recognized as by-products of the Maillard process, resulting from the Strecker degradation of the respective amino acids [39]. As for carboxylic acids, acetic, propanoic, and hexanoic acids have already been described in the volatile composition of roasted onions [36].

**Figure 2:**
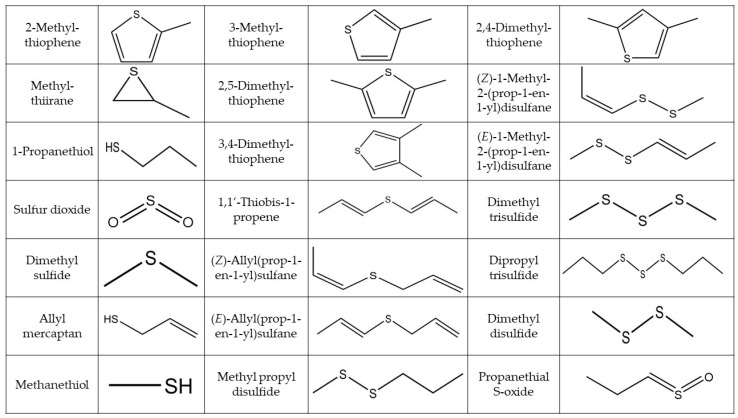
Identification of organosulfur compounds found in red onions: chemical structures.

This study focused on the twenty organosulfur compounds identified in onions (Table 2) and aimed to analyze the impact of DTD conditions on their extraction and analysis. Figure 2 shows their chemical structures, and the characteristic mass spectral ions are shown in the Supplementary Materials (Table S1).

### 2.2. Individual Box–Behnken Designs and Analysis of Variances

Two Box-Behnken designs were carried out for the development and optimisation of the DTD-GC-MS method for the pre-extraction and post-analysis of both the total sulfur compounds area and the area of the propanethial S-oxide. The ANOVA was applied to each experimental matrix, and the results are shown in Table 2 and Table 3.

The models effectively described the observed data for both responses, as well as the total area (Y_TA_) and the area of the propanethial S-oxide (Y_C3H6OS_), explaining 84.84% and 76.35% of their variability, respectively. Additionally, both models fit well as lack-of-fit tests with *p*-values greater than 0.05 (0.0577 and 0.0586, respectively). So, 2 s-order equations can be constructed to predict each response value as a function of the independent variables (Equations (1) and (2)).

Y_TA_ (g^−1^) = 7.36 × 10^9^ − 5.97 × 10^8^·X_1_ + 9.83 × 10^8^·X_2_ + 7.29 × 10^7^·X_3_ + 9.92 × 10^7^·X_4_ − 4.91E+06·X_5_ − 6.81 × 10^8^·X_1_^2^ − 1.30 × 10^8^·X_1_X_2_ + 1.88 × 10^8^·X_1_X_3_ − 1.37 × 10^7^·X_1_X_4_ − 3.59 × 10^7^·X_1_X_5_ − 4.13 × 10^8^·X_2_^2^ + 5.12 × 10^8^·X_2_X_3_ − 7.35 × 10^7^·X_2_X_4_ − 1.65 × 10^8^·X_2_X_5_ − 6.09 × 10^8^·X_3_^2^ − 1.12 × 10^8^·X_3_X_4_ + 3.62 × 10^8^·X_3_X_5_ − 5.44 × 10^8^·X_4_^2^ + 2.90 × 10^8^·X_4_X_5_ − 6.77 × 10^8^·X_5_^2^, (1)

Y_C3H6OS_ (g^−1^) = 5.26 × 10^8^ − 1.16 × 10^8^·X_1_ − 4.60 × 10^7^·X_2_ + 4.71 × 10^7^·X_3_ + 1.74 × 10^7^·X_4_ − 3.29 × 10^7^·X_5_ − 1.27 × 10^8^·X_1_^2^ + 1.22 × 10^8^·X_1_X_2_ + 3.05 × 10^7^·X_1_X_3_ − 2.01 × 10^7^·X_1_X_4_ − 4.36 × 10^7^·X_1_X_5_ − 1.78 × 10^8^·X_2_^2^ + 4.26 × 10^7^·X_2_X_3_ + 1.38 × 10^8^·X_2_X_4_ − 5.43 × 10^7^·X_2_X_5_ − 8.15 × 10^7^·X_3_^2^ − 7.83 × 10^7^·X_3_X_4_ + 9.73 × 10^6^·X_3_X_5_ − 1.38 × 10^8^·X_4_^2^ + 1.06E × 10^7^·X_4_X_5_ − 3.83 × 10^7^·X_5_^2^, (2)

**Figure 3:**
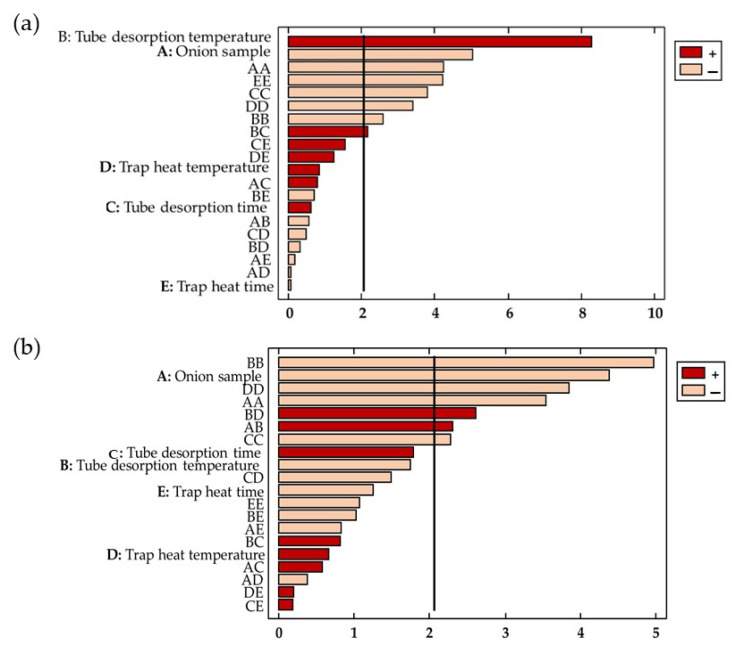
Pareto Chart of: (**a**) the total area; and (**b**) the area of the propanethial S-oxide.

Likewise, ANOVA showed the significance of each factor and its interaction with the response variable. Only variables and interactions with a *p*-value less than 0.05 were significantly affected at a 95% level of significance. This statistical information was graphically represented using the Pareto chart (Figure 3).

Onion sample weight (X_1_) significantly impacted the pre-extraction and post-analysis of the organosulfur compounds of the red onions (*p*-value < 0.05). The onion sample amount used negatively affected the total area of the organosulfur compounds (b_1_ = −5.97 × 10^8^) and the area of the propanethial S-oxide area (b_1_ = − 1.16 × 108). 

The tube desorption temperature positively affected the total area of the organosulfur compounds because greater temperatures increased the efficiency of the pre-extraction process (b_2_ = 9.83 × 10^8^). However, it is worth noting that, if the desorption temperature is too high, the peak area could show a negative trend due to the degradation of the compounds [38]. This could be observed by the effect of the desorption temperature on the area of the propanethial S-oxide—the effect was not significant, but negative. The conditions in the tube oven during the desorption stage were crucial for the effective extraction of the compounds [37].

**Figure 4:**
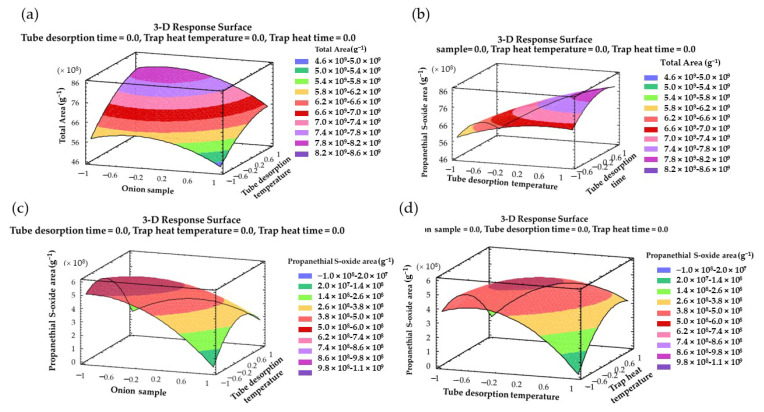
Three-dimensional (3D) surface plots of BBD using polynomial equations: (**a**) the effect of both the onion sample and the tube desorption temperature on the total organosulfur area; (**b**) the effect of both the tube desorption temperature and the tube desorption time on the total organosulfur area; (**c**) the effect of both the onion sample and the tube desorption temperature on the area of the propanethial S-oxide; and (**d**) the effect of both the tube desorption temperature and the trap heat temperature on the area of the propanethial S-oxide.

Finally, for a clear understanding of the interactive and main effects, 3D surface plots were represented using the designed model. Figure 4a–d show the combined impact of the onion sample–tube desorption temperature, the tube desorption temperature–tube desorption time, and the tube desorption temperature–trap heat temperature, on the response variables.

### 2.3. Multi-Response Optimization

Finally, RSM provided details on the optimal values that each factor should assume to achieve maximum response. Specifically, the values required to optimize the extraction of total organosulfur and the extraction of propanethial S-oxide are included in Table 4. On the other hand, to identify the best conditions not only for the total area but also for the area of the propanethial S-oxide, the MRO was applied. As Table 4 shows, the optimal conditions obtained through both individual experiments and MRO achieved a desirable value of 84.8%. 

The precision of the MRO method was also validated through conducting 27 experiments carried out on 3 consecutive days. The approach exhibited desirable reproducibility and intermediate precision, as evidenced by a CV below 10% for all organosulfur compounds (Supplementary Material, Table S2). These results were considered acceptable, as the CVs were below the commonly accepted threshold of 10% [37]. 

**Figure 5:**
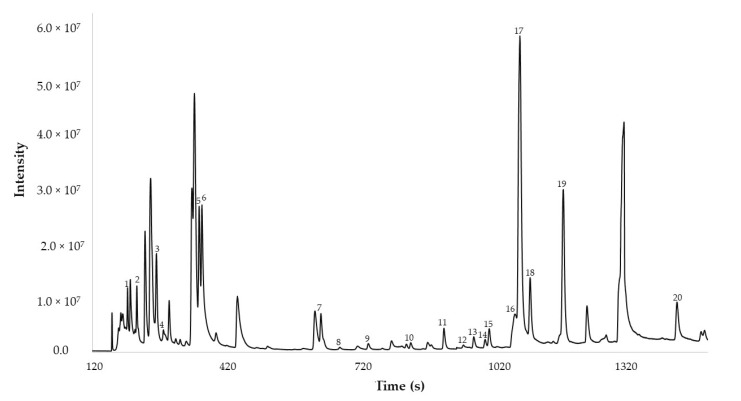
DTD-GC-MS chromatogram of the organosulfur compounds present in red onions. The code compounds are shown in Table 5.

The validated method was applied to the red onion sample, obtaining the results of the area shown in Table 5 and the total ion chromatogram (TIC) shown in Figure 5. 

### 2.4. Distribution of the Organosulfur Compound in Red Onion by MRO DTD-GC-MS Method

**Figure 6:**
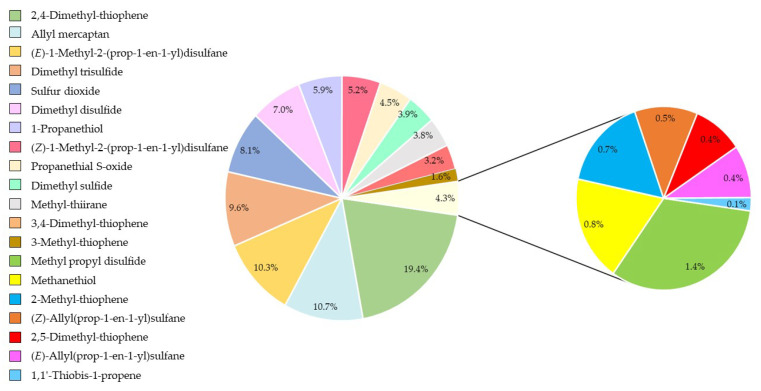
Distribution of the twenty organosulfur compounds. Each color shows its percentage composition calculated by using the normalization method from the GC peak areas.

The optimized method for analyzing the distribution of 20 sulfur compounds in a red onion sample was then used, and the results are included in Figure 6.

When alliinase and cysteine sulfoxides came together, they generated a mixture of sulfenic acids, ammonia, and pyruvate. The major onion cysteine sulfoxide, i.e., S-1-propenyl-L-cysteine sulfoxide, was transformed into the 1-propenyl sulfenic acid, which was then turned into propanethial S-oxide. This organosulfur compound, which is known as the onion lachrymatory factor (LF), accounted for 4.5% of the composition of red onions and was stable during the gas chromatography analysis, making it easy to trap and measure.

The other products of the condensation reaction, i.e., thiosulfinates, were degraded during trapping and GC analysis, thus generating most of the organosulfur compounds identified, i.e., the sulfides [39], which were involved in further transformations and showed biological activity [34]. Disulfides were observed in frozen onions, while the drying of onions increased trisulfides [40]. The resulting mixture of monosulfides, disulfides, and trisulfides accounted for 4.9%, 23.9%, and 9.6% (i.e., 38.4% of carbon sulfide CS_n_) of the red onion composition, respectively. Additionally, propanothiol was identified as a significant source of flavor in fresh onions [41], accounting for 5.9% of the red onion composition.

In addition, high temperatures during thermal desorption can trigger the thermolysis of alkyl-1-propenyl disulphides and di(1-alkenyl) disulphides to form thiophene [42]. This family of compounds accounted for 25.3% of the red onion composition, so it was one of the main components.

### 2.5. Comparison of DTD Methodology with Another Extraction Techniques

Finally, the developed MRO DTD-GC-MS method was compared with other extraction techniques to highlight its advantages.

Concerning solvent extraction techniques for volatile compounds, DTD offers multiple advantages as reported by other authors [34,43]. Firstly, it is an automated extraction technique in which there is hardly any sample handling, and it does not use solvents, which means that it is a more environmentally friendly technique. In addition, the yields and the sensitivity offered by this technique are much higher than conventional solvent extractions. 

However, it should be noted that it is not the only solvent-free technique currently available in the literature. SPME combined with HS has been used on several occasions for the study of sulfur compounds in onion samples [8,43]. Nonetheless, the choice of the adsorbent material is consistently a crucial aspect of the process because it must adsorb a diverse range of molecules with varying chemical properties, molecular weights, and polarities. With SPME, the fiber’s surface capacity is relatively restricted, which can lead to analytes competing for adsorption sites, potentially causing a greater bias towards specific volatile compounds [18]. Overall, TD typically has a higher surface capacity than SPME due to the larger size of the trap used in TD compared to the SPME fiber. To facilitate an experimental comparison of both techniques, an analysis of the organosulfur compounds in the same onion matrix using SPME (AOC-6000 Plus Multifunctional Autosampler, Shimadzu, Kyoto, Japan) was carried out. The SPME analysis conditions used were as follows: 0.5 g of onion was pre-incubated at 150 °C with pulsed agitation for 10 min at a speed of 500 rpm. The headspace above the samples in a vial was exposed to a DVB/Carbon WR/PDMS SPME fiber (manufactured by Shimadzu, Kyoto, Japan) at a depth of 22 mm for 20 min. After extraction, the SPME fiber was withdrawn and introduced into the gas chromatograph inlet, where it was desorbed for 5 min using a split mod at a temperature of 220 °C. The results obtained showed that the number of sulfur compounds identified by SPME (14 sulfur compounds) was lower than that identified by the MRO DTD-GC-MS method (20 sulfur compounds). In addition, the extraction of these compounds also showed worse results, with a total area by SPME of 78,595,746 ± 612,8231 g^−1^ —lower than the total area obtained by DTD (9,389,028,511 ± 908,282,182 g^−1^). This shows that, in the case of sulfur compounds in onion, DTD correctly optimized by BBD and MRO and combined with GC-MS provides a suitable extraction and analysis method for the compounds of interest, yielding better results than other more common techniques, such as SPME.

## 3. Materials and Methods

### 3.1. Onion Samples

The onion samples that were the focus of this investigation were procured from a nearby marketplace located in the province of Cadiz, Spain. Specifically, this variety has been used by the research group in previous studies about the evaluation of the content of phenolic compounds [9,10,11,44]. For all the experiments carried out, the onions used were in freeze-dried form to avoid the presence of water and to have a more homogeneous onion matrix [45]. The type of onion used for our investigation is a variety of Spanish origin that is cultivated from June to December. The bulbs were characterized by their globose/conical shape with red outer skins in different shades (depending on the variety). The flesh has an intense purple color, a strong taste, and high pungency.

### 3.2. DTD-GC-MS Procedure and Conditions

The DTD equipment used was a TD-20 System (Shimadzu, Kyoto, Japan). Specifically, the onion sample was placed in a sample tube (with an outer diameter of 1/4′′ (6.35 mm) and a length of 90 mm) and was secured at both ends with silica wool to prevent leakage. The flow rate of the carrier gas (helium) was adjusted to 1 mL s^−1^. The cold trap, which concentrated the desorbed compounds to a bandwidth compatible with the capillary column, was set to −15 °C. The compounds were injected into the GC module in a split mode with a split ratio of 1:50. The sample amount collected in the tube, the temperature and time of the sample-tube heating block, and the temperature and time of the trap desorption were chosen according to the results of the response surface design of experiments. A detailed outline of the process is shown in the Supplementary Materials (Figure S1). 

The GC-MS equipment used was GCMS-TQ8040 (Shimadzu, Kyoto, Japan). The chromatographic separation was performed on a Suprawax-280 capillary column (Teknokroma, Barcelona, Spain; 60 m length × 0.25 mm column I.D. × 0.25 µm film thickness). The injector was set at 25 °C. Moreover, the temperature program of the oven was as follows: 40 °C isothermal for 300 s; from 40 °C to 200 °C at a rate of 0.05 °C s^−1^; 200 °C isothermal for 300 s; from 200 °C to 270 °C at a rate of 0.67 °C s^−1^; and 270 °C isothermal for 120 s. Likewise, helium (99.999%) was used as the carrier gas at both a constant linear velocity of 35 cm s^−1^ and a flow rate of 0.031 mL s^−1^. Regarding the mass spectrometer, the ionization mode was electron impact with a voltage of 70 eV. The mass spectrometer worked in a full-scan mode in the range of 40–400 *m*/*z*. The ion source temperature was 200 °C.

Compounds were identified by comparing their mass spectra using the Wiley library (Wiley Registry of Mass Spectral Data, 7th Edition, 2000) and the criterion of at least 80% similarity [46]. The area of the chromatographic signal produced by the largest mass fragment (base peak) was measured to determine the area of each compound. Furthermore, a normalizing approach was used to obtain the percentage composition (%) from the peak area of each compound: the area of the base peak/total area.

### 3.3. Box–Behnken Design

The parameters that affected the analysis of the organosulfur compounds by using DTD-GC-MS were optimized with a BBD [47]. As aforementioned, several factors were evaluated to determine their optimum levels: the onion sample amount placed in the sample tube (X_1_), the tube desorption temperature (X_2_), the tube desorption time (X_3_), the trap heat temperature (X_4_), and the trap heat time (X_5_). In a BBD, each factor was studied at 3 levels: at a lower level (−1), at an intermediate level (0), and at a higher level (1). Due to the lack of prior studies on the application of DTD to analyze the organosulfur compounds in onions, the operating range of each factor for the BBD was selected according to the outcome of the OFAT experiments. The OFAT experiments are summarized in the Supplementary Materials (Table S3 and Figure S2). According to these results, the range for each factor was chosen to consider not only the greatest total areas but also the area of the individual propanethial S-oxide: 40-50-60 mg for X_1_; 180-200-220 °C for X_2_; 600-900-1200 s for X_3_; 250-265-280 °C for X_4_; 120-180-240 s for X_5_. Two response variables were defined: the total area (Y_TA_) of the base peak of each compound, and the area of the propanethial S-oxide (Y_C3H6OS_). The latter is of interest as it is obtained from the main SCs of onions and plays the role of lacrimator [48]. Both response variables were expressed as relative areas, i.e., as a function of the accurate mass of onion weighed for each experiment: the total area per gram of onion (g^−1^) and the area of the propanethial S-oxide per gram of onion (g^−1^), respectively. The study involved two separate BBD experiments, each design consisted of 46 treatments, including 6 repetitions at the center point to calculate the error. The complete matrix with the experimental and predicted values for each response variable is included in Supplementary Materials (Table S4).

### 3.4. Response Surface Methodology

In combination with BBD, RSM comprises a series of statistical and mathematical methods utilized to develop and optimize processes. RSM allows for the modelling of the curvature relationship between factors and their response by employing a second-order polynomial equation (Equation (3)) [49].

(3) $$Y=\beta_{0}+\sum_{i=1}^{k}\beta_{i}X_{i}+\sum_{i=1}^{k}\;\beta_{ii\;}X_{i}^{2}+\sum \sum_{i<j}^{k}\beta_{ij}X_{i}X_{j}+r\;,$$

where *Y* represents the predicted responses (i.e., *Y_TA_* and *Y_C3H6OS_*); *X_i_* and *X_j_* the factors involved; *X_i_X_j_* the interactions between factors; *X_i_^2^* the quadratic interaction between factors; *β_0_* the intercept; *β_i_* the linear coefficient; *β_ij_*_,_ the coefficient of interaction between factors; *β_ii_* the quadratic coefficient; and *r* the random error. 

Utilizing the Statgraphics Centurion version XVI software (Warrenton, VA, USA) and Design Expert software (Version 13, Stat-Ease Inc., Minneapolis, MN, USA), an analysis of variance (ANOVA) was executed.

After optimizing each of the two response variables separately, a multi-response optimization (MRO) was performed to determine their optimal conditions simultaneously. The desirability optimization methodology was employed, which combines the desirability function analysis with the design of experiments [50]. Each response was assigned a desirability score (d_i_) ranging from 0 to 1, where 0 indicated an unacceptable response, and 1 indicated an ideal response. The individual desirability scores were then geometrically averaged to obtain an overall desirability score (D). Ultimately, the multi-optimization process aimed to maximize the value of D. 

## 4. Conclusions

Among all vegetables, onions have perhaps the biggest market niche not only because of their great use in cooking but also because of their sulfur compounds, which can have enormously beneficial properties for one’s health. Particularly for the latter reason, it is necessary to develop methods of analysis and extraction that allow for the study of these sulfur compounds in an efficient way. In this work, a DTD-GC-MS methodology has been developed for the pre-extraction and subsequent determination of the organosulfur compounds present in onions. A BBD, together with MRO, was used for optimization, considering the total area (sum of the individual area of each of the 20 identified sulfur compounds) and the individual area of the propanethial S-oxide as response variables. The MRO conditions were as follows: 46 mg of onion in the tube, a desorption heat of 205 °C for 960 s, and a trap heat of 267 °C for 180 s. In addition, the efficacy of the technique has been confirmed by demonstrating that all organosulfur compounds exhibit high levels of repeatability and intermediate precision, with coefficients of variation (CV) lower than 10%. The distribution of the twenty organosulfur compounds showed 25.3% of thiophenes, 38.4% of carbon sulfides (mixture of monosulfides, disulfides, and trisulfides), and 4.5% of propanethial S-oxide. Overall, the optimized DTD-GC-MS method has significant practical implications for laboratories, researchers, and companies hoping to determine the organosulfur content accurately and reliably in onions. To evaluate the limitations of this study, the developed method was compared with SPME, the most widely used pre-extraction technique, to study these compounds in onions. The developed method showed better extractions, with a higher amount of extracted sulfur compounds. In the future, high-resolution analytical techniques will allow us to know more about the content of these compounds in different types of onions, which could be useful for assessing how factors such as variety, origin, and cultivation method affect the composition and activity of these compounds.

## Figures and Tables

**Table 1:** First qualitative analyses of the lyophilized red onion by using DTD-GC-MS.

Name	Formula	N° CAS	Similarity (%)
**Carboxylic acid**			
Acetic acid	C_2_H_4_O_2_	64-19-7	97
Formic acid	CH_2_O_2_	64-18-6	98
Propanoic acid	C_3_H_6_O_2_	79-9-4	83
**Ester**			
Oxiranylmethyl ester 2-propenoic acid	C_6_H_8_O_3_	106-90-1	90
Methyl ester hexadecanoic acid	C_17_H_34_O_2_	112-39-0	90
Ethyl ester 2-methyl-3-oxo-butanoic acid	C_7_H_12_O_3_	609-14-3	83
2-Hydroxy-*gamma*-butyrolactone	C_4_H_6_O_3_	19444-84-9	87
3,5-Dihydroxy-2-methyl-4H-pyran-4-one	C_6_H_6_O_4_	1073-96-7	92
**Alkane**			
1-(1H-pyrrol-2-yl)-ethanone	C_6_H_7_NO	1072-83-9	94
**Alcohol**			
Ethanol	C_2_H_6_O	64-17-5	98
2,3-Butanediol	C_4_H_10_O_2_	513-85-9	92
3-Furanmethanol	C_5_H_6_O_2_	4412-91-3	94
2-Furanmethanol	C_5_H_6_O	98-0-0	90
5-Methyl-2-furan methanol	C_6_H_8_O_2_	3857-25-8	95
3-Butene-1,2-diol	C_4_H_8_O_2_	497-6-3	88
**Aldehydes**			
Acetaldehyde	C_2_H_4_O	75-7-0	98
Propanal	C_3_H_6_O	123-38-6	96
2-Methyl-propanal	C_4_H_8_O	78-84-2	98
2-Methyl-butanal	C_5_H_10_O	96-17-3	96
2-Methyl-2-butenal	C_5_H_10_O	590-86-3	93
2-Methyl-2-pentenal	C_6_H_10_O	14250-96-5	96
Furfural	C_5_H_4_O_2_	98-1-1	98
2,2-Diethylbutyraldehyde	C_8_H_16_O	26254-89-7	85
**Ketones**			
2-Butanone	C_4_H_8_O	78-93-3	86
2,3-Butanedione	C_4_H_6_O_2_	431-03-8	96
Acetoin	C_4_H_8_O_2_	513-86-0	90
1-Hydroxy-2-propanone	C_3_H_6_O	116-9-6	98
4,5-Dimethyl-1,3-dioxol-2-one	C_5_H_6_O_3_	37830-90-3	86
**Furans**			
3-Methyl-furan	C_5_H_6_O	930-27-8	95
2,4-Dimethylfuran	C_6_H_8_O	3710-43-8	95
**Other compounds without S**			
Carbon dioxide	CO_2_	124-38-9	98
**Organosulfur compounds**			
Thiols			
Methanethiol	CH_4_S	74-93-1	98
1-Propanethiol	C_3_H_8_S	107-3-9	95
Allyl mercaptan	C_3_H_6_S	870-23-5	94
**Monosulfide**			
Dimethyl sulfide	C_2_H_6_S	75-18-3	98
1,1’-Thiobis-1-propene	C_6_H_10_S	33922-80-4	89
(*Z*)-Allyl(prop-1-en-1-yl)sulfane *	C_6_H_10_S	104324-69-8	87
(*E*)-Allyl(prop-1-en-1-yl)sulfane *	C_6_H_10_S	104324-36-9	85
**Disulfides**			
Dimethyl disulfide	C_2_H_6_S_2_	624-92-0	95
Methyl propyl disulfide	C_4_H_10_S_2_	2179-60-4	93
(*Z*)-1-Methyl-2-(prop-1-en-1-yl)disulfane *	C_4_H_8_S_2_	23838-19-9	96
(*E*)-1-Methyl-2-(prop-1-en-1-yl)disulfane *	C_4_H_8_S_2_	23838-18-8	97
**Trisulfide**			
Dimethyl trisulfide	C_2_H_6_S_3_	3658-80-8	97
**Episulfide**			
Methyl-thiirane	C_3_H_6_S	1072-43-1	94
**Thiophene**			
2-Methyl-thiophene	C_5_H_6_S	554-14-3	93
3-Methyl-thiophene	C_5_H_6_S	616-44-4	96
2,5-Dimethyl-thiophene	C_6_H_8_S	638-02-8	91
3,4-Dimethyl-thiophene	C_6_H_8_S	638-0-6	94
2,4-Dimethyl-thiophene	C_6_H_8_S	638-00-6	95
**Sulfine**			
Propanethial S-oxide	C_3_H_6_OS	32157-29-2	96
**Other S-Compounds**			
Sulfur dioxide	SO_2_	7446-09-5	96

* The identification of the isomer is tentative.

**Table 2:** ANOVA of the quadratic model for the total area.

Source	Source Code	Coefficients	Sum of Squares	DF	Mean Square	*F*-Value	*p*-Value
A: Onion sample	X_1_	−5.97 × 10^8^	5.70 × 10^18^	1	5.70 × 10^18^	91.32	<0.001
B: Tube desorption temperature	X_2_	9.83 × 10^8^	1.55 × 10^19^	1	1.55 × 10^19^	247.71	<0.001
C: Tube desorption time	X_3_	7.29 × 10^7^	8.50 × 10^16^	1	8.50 × 10^16^	1.36	0.296
D: Trap heat temperature	X_4_	9.92 × 10^7^	1.57 × 10^17^	1	1.57 × 10^17^	2.52	0.173
E: Trap heat time	X_5_	−4.91 × 10^6^	3.86 × 10^14^	1	3.86 × 10^14^	0.01	0.940
AA	X_1_^2^	−6.81 × 10^8^	4.05 × 10^18^	1	4.05 × 10^18^	64.89	<0.001
AB	X_1_X_2_	−1.30 × 10^8^	6.77 × 10^16^	1	6.77 × 10^16^	1.08	0.345
AC	X_1_X_3_	1.88 × 10^8^	1.42 × 10^17^	1	1.42 × 10^17^	2.27	0.192
AD	X_1_ X_4_	−1.37 × 10^7^	7.52 × 10^14^	1	7.52 × 10^14^	0.01	0.917
AE	X_1_X_5_	−3.59 × 10^7^	5.15 × 10^15^	1	5.15 × 10^15^	0.08	0.786
BB	X_2_^2^	−4.13 × 10^8^	1.49 × 10^18^	1	1.49 × 10^18^	23.8	0.00460
BC	X_2_X_3_	5.12 × 10^8^	1.05 × 10^18^	1	1.05 × 10^18^	16.78	0.00940
BD	X_2_X_4_	−7.35 × 10^7^	2.16 × 10^16^	1	2.16 × 10^16^	0.35	0.582
BE	X_2_X_5_	−1.65 × 10^8^	1.08 × 10^17^	1	1.08 × 10^17^	1.74	0.245
CC	X_3_^2^	−6.09 × 10^8^	3.24 × 10^18^	1	3.24 × 10^18^	51.89	<0.001
CD	X_3_X_4_	−1.12 × 10^8^	4.98 × 10^16^	1	4.98 × 10^16^	0.8	0.413
CE	X_3_X_5_	3.62 × 10^8^	5.25 × 10^17^	1	5.25 × 10^17^	8.41	0.0338
DD	X_4_^2^	−5.44 × 10^8^	2.58 × 10^18^	1	2.58 × 10^18^	41.4	0.00130
DE	X_4_X_5_	2.90 × 10^8^	3.37 × 10^17^	1	3.37 × 10^17^	5.4	0.0678
EE	X_5_^2^	−6.77 × 10^8^	4.00 × 10^18^	1	4.00 × 10^18^	64.12	<0.001
Lack-of-fit			5.31 × 10^18^	20	2.65 × 10^17^	4.25	0.0577
Residual			5.62 × 10^18^	25	2.25 × 10^17^		
Pure Error			3.12 × 10^17^	5	6.24 × 10^16^		
Cor Total			3.71 × 10^19^	45			
Model		7.36 × 1009					

**Table 3:** ANOVA of the quadratic model for the area of the propanethial S-oxide.

Source	Source Code	Coefficients	Sum of Squares	DF	Mean Square	*F*-Value	*p*-Value
A: Onion sample	X_1_	−1.16 × 10^8^	2.15 × 10^17^	1	2.15 × 10^17^	68.8	0.0004
B: Tube desorption temperature	X_2_	−4.60 × 10^7^	3.39 × 10^16^	1	3.39 × 10^16^	10.83	0.0217
C: Tube desorption time	X_3_	4.71 × 10^7^	3.55 × 10^16^	1	3.55 × 10^16^	11.33	0.0200
D: Trap heat temperature	X_4_	1.74 × 10^7^	4.86 × 10^15^	1	4.86 × 10^15^	1.55	0.268
E: Trap heat time	X_5_	−3.29 × 10^7^	1.73 × 10^16^	1	1.73 × 10^16^	5.53	0.0655
AA	X_1_^2^	−1.27 × 10^8^	1.40 × 10^17^	1	1.40 × 10^17^	44.6	0.00110
AB	X_1_X_2_	1.22 × 10^8^	5.92 × 10^16^	1	5.92 × 10^16^	18.89	0.00740
AC	X_1_X_3_	3.05 × 10^7^	3.72 × 10^15^	1	3.72 × 10^15^	1.19	0.325
AD	X_1_X_4_	−2.01 × 10^7^	1.61 × 10^15^	1	1.61 × 10^15^	0.52	0.505
AE	X_1_X_5_	−4.36 × 10^7^	7.62 × 10^15^	1	7.62 × 10^15^	2.43	0.180
BB	X_2_^2^	−1.78 × 10^8^	2.77 × 10^17^	1	2.77 × 10^17^	88.41	<0.001
BC	X_2_X_3_	4.26 × 10^7^	7.27 × 10^15^	1	7.27 × 10^15^	2.32	0.188
BD	X_2_X_4_	1.38 × 10^8^	7.61 × 10^16^	1	7.61 × 10^16^	24.28	0.00440
BE	X_2_X_5_	−5.43 × 10^7^	1.18 × 10^16^	1	1.18 × 10^16^	3.76	0.110
CC	X_3_^2^	−8.15 × 10^7^	5.80 × 10^16^	1	5.80 × 10^16^	18.53	0.00770
CD	X_3_X_4_	−7.83 × 10^7^	2.45 × 10^16^	1	2.45 × 10^16^	7.83	0.0381
CE	X_3_X_5_	9.73 × 10^6^	3.79 × 10^14^	1	3.79 × 10^14^	0.12	0.742
DD	X_4_^2^	−1.38 × 10^8^	1.66 × 10^17^	1	1.66 × 10^17^	52.95	<0.001
DE	X_4_X_5_	1.06 × 10^7^	4.47 × 10^14^	1	4.47 × 10^14^	0.14	0.721
EE	X_5_^2^	−3.83 × 10^7^	1.28 × 10^16^	1	1.28 × 10^16^	4.09	0.0992
Lack-of-fit			2.64 × 10^17^	20	1.32 × 10^16^	4.21	0.0586
Residual			2.80 × 10^17^	25	1.12 × 10^16^		
Pure Error			1.57 × 10^16^	5	3.13 × 10^15^		
Cor Total			1.18 × 10^18^	45			
Model		5.26 × 10^8^					

**Table 4:** Results of the individual optimization of the total area and the area of the propanethial S-oxide, as well as MRO of both response variables.

Factors	Individual Optimization		MRO
	Total Area	Propanethial S-Oxide Area	
X_1_: Onion sample (mg)	46	47	46
X_2_: Tube desorption temperature (°C)	211	189	205
X_3_: Tube desorption time (s)	16	9	16
X_4_: Trap heat temperature (°C)	265	252	267
X_5_: Trap heat time (s)	3	2	3

**Table 5:** Organosulfur compounds extracted and analyzed using the DTD-GC-MS MRO optimized method. Compositional percentages were computed by the normalization method from the GC peak areas.

Code	Compound	Individual Relative Area (g^−1^)	Percentage Composition (%)
1	Methanethiol	79,946,258 ± 5,037,254	0.8 ± 0.1
2	Dimethyl sulfide	368,819,241 ± 26,379,719	3.9 ± 0.3
3	1-Propanethiol	520,814,238 ± 25,833,947	5.9 ± 1.2
4	Sulfur dioxide	741,589,894 ± 68,150,056	8.1 ± 0.3
5	Methyl-thiirane	356,083,962 ± 6,570,787	3.8 ± 0.2
6	Allyl mercaptan	925,374,280 ± 53,675,232	10.7 ± 0.6
7	Dimethyl disulfide	734,818,197 ± 68,447,885	7.0 ± 0.4
8	2-Methyl-thiophene	79,399,050 ± 7,255,625	0.7 ± 0.04
9	3-Methyl-thiophene	161,784,317 ± 15,999,654	1.6 ± 0.1
10	2,5-Dimethyl-thiophene	46,771,965 ± 4,109,263	0.4 ± 0.03
11	3,4-Dimethyl-thiophene	266,951,634 ± 26,402,475	3.2 ± 0.3
12	1,1’-Thiobis-1-propene	12,137,629 ± 982,646	0.1 ± 0.01
13	(*Z*)-Allyl(prop-1-en-1-yl)sulfane	47,080,601 ± 3,942,928	0.5 ± 0.03
14	(*E*)-Allyl(prop-1-en-1-yl)sulfane	34,336,629 ± 1,522,835	0.4 ± 0.03
15	Methyl propyl disulfide	127,532,708 ± 1,832,161	1.4 ± 0.1
16	Propanethial S-oxide	385,303,062 ± 14,922,610	4.5 ± 0.3
17	2,4-Dimethyl-thiophene	1,728,974,335 ± 94,208,493	19.4 ± 0.1
18	(*Z*)-1-Methyl-2-(prop-1-en-1-yl)disulfane	457,771,521 ± 11,134,133	5.2 ± 0.5
19	(*E*)-1-Methyl-2-(prop-1-en-1-yl)disulfane	983,648,059 ± 82,014,566	10.3 ± 0.4
20	Dimethyl trisulfide	839,379,417 ± 73,906,935	9.6 ± 1.0
	Total	9,389,028,511 ± 908,282,182	-

## Data Availability

The data presented in this study are contained within the article.

## Supplementary Materials

The following supporting information can be downloaded at: https://www.mdpi.com/article/10.3390/ph16050715/s1, Figure S1: Schematic diagram of the DTD system; Figure S2: OFAT experiments concerning the effect of the factor: (a) onion sample amount placed in the sample tube (X_1_), (b) tube desorption temperature (X_2_), (c) tube desorption time (X_3_), (d) trap heat temperature (X_4_), and (e) trap heat time (X_5_), on both the relative total area (g^−1^) and the relative area of the propanethial S-oxide (g^−1^); Table S1: Characteristic mass spectral ions of the organosulfur compounds identified in red onion; Table S2: Validation of the developed DTD-GC-MS MRO method; Table S3: OFAT experiments to choose the range of BBD (*n* = 3); Table S4: BBD for the total area and the area of the propanethial S-oxide. The results corresponded to experimental and predicted values.
