# Supplementary material for: Application of Direct Thermal Desorption–Gas Chromatography–Mass Spectrometry for Determination of Volatile and Semi-Volatile Organosulfur Compounds in Onions: A Novel Analytical Approach
Source: Pharmaceuticals (Basel). 2023 May 8;16(5):715. doi: 10.3390/ph16050715 (PMC10220549; doi:10.3390/ph16050715)
Supplement: Supplementary file 1 [file pharmaceuticals-16-00715-s001.zip › pharmaceuticals-2297621-supplementary.pdf]

SUPPLEMENTARY MATERIAL

Table S1. Characteristic mass spectral ions of the organosulfur compounds identified in red onion.

Peak nº	Compound	Base peak *m/z* (100%)	Characteristic mass spectral ions *m/z* (relativity intensity)
1	Methanethiol	47	48 (92), 45 (46)
2	Dimethyl sulfide	62	47 (90), 46 (38)
3	1-Propanethiol	76	47 (79), 42 (70)
4	Sulfur dioxide	64	48 (42), 56 (8)
5	Methyl-thiirane	74	41 (83), 45 (60)
6	Allyl mercaptan	74	41 (85), 45 (70)
7	Dimethyl disulfide	94	79 (42), 45 (33)
8	2-Methyl-thiophene	97	98 (58), 45 (9)
9	3-Methyl-thiophene	97	98 (58), 45 (11)
10	2,5-Dimethyl-thiophene	111	112 (94), 97 (57)
11	3,4-Dimethyl-thiophene	111	112 (87), 97 (48)
12	1,1'-Thiobis-1-propene	99	114 (91), 41 (63)
13	(*Z*)-Allyl(prop-1-en-1-yl)sulfane	99	114 (92), 45 (59)
14	(*E*)-Allyl(prop-1-en-1-yl)sulfane	114	99 (94), 45 (60)
15	Methyl propyl disulfide	80	122 (85), 43 (53)
16	Propanethial S-oxide	41	90 (48), 42 (30)
17	2,4-Dimethyl-thiophene	97	112 (99.9), 111 (99.7)
18	(*Z*)-1-Methyl-2-(prop-1-en-1-yl)disulfane	120	45 (59), 72 (48)
19	(*E*)-1-Methyl-2-(prop-1-en-1-yl)disulfane	120	45 (45), 72 (48)
20	Dimethyl trisulfide	126	79 (40), 45 (36)

Table S2. Validation of the developed DTD-GC-MS MRO method.

Compound	Repeatability, CV(%)	Intermediate precision, CV (%)
Methanethiol	6.3	7.7
Dimethyl sulfide	7.2	8.5
1-Propanethiol	5.0	8.0
Sulfur dioxide	8.9	9.2
Methyl-thiirane	1.8	5.8
Allyl mercaptan	3.0	5.8
Dimethyl disulfide	9.1	9.3
2-Methyl-thiophene	2.9	9.3
3-Methyl-thiophene	8.6	9.9
2,5-Dimethyl-thiophene	8.8	9.4
3,4-Dimethyl-thiophene	9.9	9.9
1,1'-Thiobis-1-propene	8.1	8.5
(*Z*)-Allyl(prop-1-en-1-yl)sulfane	5.1	8.4
(*E*)-Allyl(prop-1-en-1-yl)sulfane	4.0	4.4
Methyl propyl disulfide	1.4	6.8
Propanethial S-oxide	3.7	3.9
2,4-Dimethyl-thiophene	5.4	8.1
(*Z*)-1-Methyl-2-(prop-1-en-1-yl)disulfane	2.4	9.2
(*E*)-1-Methyl-2-(prop-1-en-1-yl)disulfane	8.3	9.2
Dimethyl trisulfide	8.9	9.8
Total	9.3	9.7

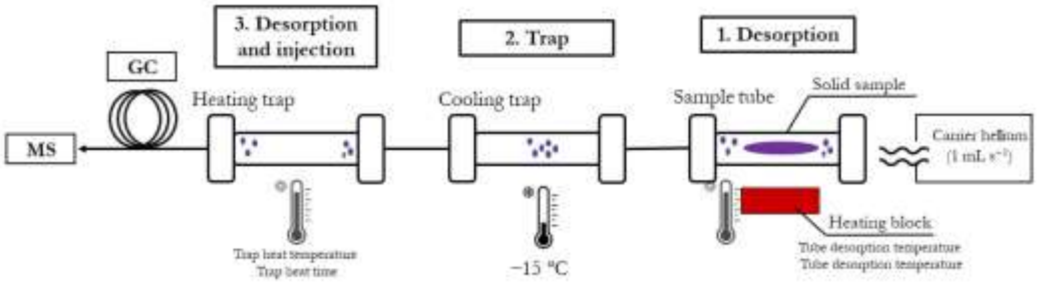

Figure S1. Schematic diagram of the DTD system.

Table S3. OFAT experiments to choose the range of BBD (n = 3).

	X_1 (mg)	X_2 (°C)	X_3 (s)	X_4 (°C)	X_5 (s)
Exp. 1	10, 20, 30, 40, 50, 60	150	600	265	180
Exp. 2	50	120, 150, 180, 200, 220, 240	600	265	180
Exp. 3	50	150	300, 600, 900, 1320	265	180
Exp. 4	50	150	600	250, 265, 280	180
Exp. 5	50	150	600	265	120, 180, 240, 300, 360

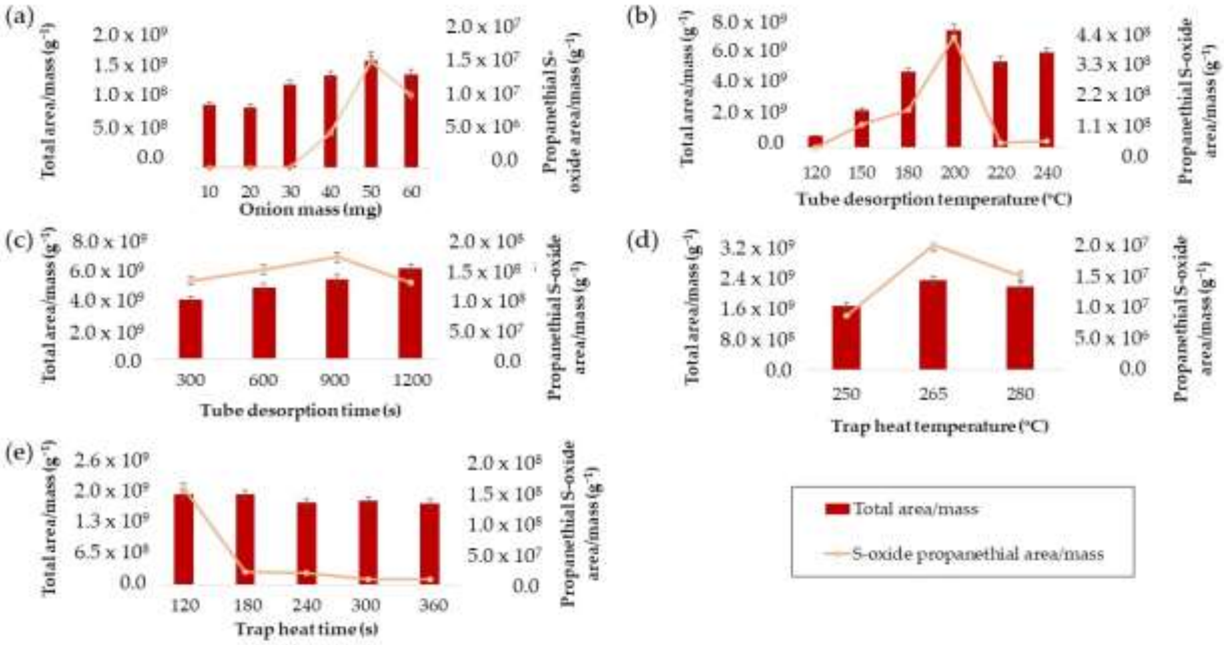

Figure S2. OFAT experiments concerning the effect of the factor: (a) onion sample amount placed in the sample tube (X_1), (b) tube desorption temperature (X_2), (c) tube desorption time (X_3), (d) trap heat temperature (X_4), and (e) trap heat time (X_5), on both the relative total area (g^{-1}) and the relative area of the propanethial S-oxide (g^{-1}).

Table S4. BBD for the total area and the area of the propanethial S-oxide. The results corresponded to experimental and predicted values.

Run	Factors					Responses			
	X_1	X_2	X_3	X_4	X_5	Y_{TA} (g^{-1})[1]		Y_{C3H6OS} (g^{-1})[1]	
						**Experimental**	**Predicted**	**Experimental**	**Predicted**
1	0	0	−1	−1	0	5700758557	5921550000	208087000	163298000
2	0	−1	0	−1	0	5459456362	5246040000	465027000	376045000
3	−1	0	0	0	1	6369343674	6627880000	472342000	487506000
4	1	0	0	0	−1	5748567354	5444020000	277923000	321193000
5	1	0	−1	0	0	5379234697	5210200000	103919000	123818000
6	−1	−1	0	0	0	5570291851	5748390000	354968000	504579000
7	0	0	0	−1	1	6003523314	5742940000	275995000	288482000
8	0	0	−1	1	0	6263649848	6342950000	294816000	354773000
9	0	0	1	0	−1	4646925618	5787740000	490594000	475916000
10	0	0	1	−1	0	6083576444	6290360000	515258000	414081000
11	0	1	−1	0	0	6561076355	6735150000	2072160	130086000
12	1	0	0	0	1	5100749952	5362460000	38951400	168134000
13	0	0	0	1	−1	6097817085	5951090000	322888000	389114000
14	1	0	1	0	0	6058741987	5732190000	281449000	278997000
15	1	0	0	−1	0	4839520917	5450790000	52343600	147766000
16	−1	0	1	0	0	6874299904	6549620000	524642000	450071000
17	0	0	0	1	1	6693685615	6521570000	321305000	344494000
18	0	0	0	−1	−1	6568249040	6333060000	319884000	375409000
19	1	−1	0	0	0	4960655161	4814890000	92315700	29258400
20	−1	1	0	0	0	7585769027	7974550000	144624000	169264000
21	1	0	0	1	0	5613682276	5621690000	176685000	142450000
22	1	1	0	0	0	6455777940	6520690000	368441000	180412000
23	−1	0	−1	0	0	6947300901	6780140000	469137000	416917000
24	−1	0	0	1	0	7157352145	6842800000	475477000	414710000
25	−1	0	0	0	−1	6873691619	6565960000	536742000	465994000
26	0	−1	1	0	0	5245591315	4914910000	272543000	316333000
27	0	0	−1	0	1	6408739923	5632180000	377210000	315976000
28	0	1	0	0	1	6590342313	7082100000	269495000	175921000
29	0	−1	0	0	1	5403231460	5445500000	318080000	376509000
30	0	1	0	0	−1	7374498495	7421300000	469866000	350201000
31	0	0	1	0	1	6347258681	6502230000	513241000	429599000
32	0	1	0	1	0	7372405556	7410350000	301990000	318822000
33	0	1	1	0	0	8490075966	7904160000	80363600	309524000
34	0	1	0	−1	0	7977411298	7359060000	5566850	8189770
35	0	−1	0	0	−1	5528621525	5125940000	301437000	333776000
36	0	0	−1	0	−1	6157041198	6366320000	393476000	401206000
37	0	0	1	1	0	6200332000	6265610000	288752000	292322000
38	−1	0	0	−1	0	6328354614	6617050000	270788000	339679000
39	0	−1	0	1	0	5148525854	5591400000	209902000	135129000
40	0	−1	−1	0	0	5363140936	5792450000	364796000	307439000
41	0	0	0	0	0	7151686643	7358460000	585730000	525513000
42	0	0	0	0	0	7364662539	7358460000	437871000	525513000
43	0	0	0	0	0	7478579340	7358460000	581186000	525513000
44	0	0	0	0	0	7042831764	7358460000	538516000	525513000
45	0	0	0	0	0	7752440218	7358460000	515661000	525513000
46	0	0	0	0	0	7360558822	7358460000	494112000	525513000

[1]Area values are relative, expressed as a function of the accurate onion mass weighed for each experiment.
